# The role of mitochondrial dysfunction, oxidative stress, and gender in cardiac fibrosis and vascular remodeling in an induced aged rat model with possible mitigation by eugenol nano-emulsion

**DOI:** 10.1007/s00210-026-05262-4

**Published:** 2026-04-13

**Authors:** Rawan H. Hanafi, Marwa S. Khattab, Sara M. Baraka, Saber Ibrahim, Samar H. Elsharkawy, Reda M. S. Korany

**Affiliations:** 1 Pathology Department, Faculty of Veterinary Medicine, Cairo University, Giza, Egypt; 2 Chemistry of Natural Compounds Department, National Research Centre, Giza, 12622 Dokki Egypt; 3 Packaging Materials Department, National Research Centre, Giza, 12622 Egypt; 4 Nanomaterials Investigation Lab., Central Laboratory Network, National Research Centre, Giza, 12622 Egypt; 5 Department of Surgery, Anaesthesiology, and Radiology, Faculty of Veterinary Medicine, Cairo University, Giza, 12211 Egypt; 6 Pathology Department, Faculty of Veterinary Medicine, Egyptian Chinese University, Cairo, Egypt

**Keywords:** Aging, Histopathology, Mitophagy, Eugenol, Nanoemulsion

## Abstract

Cardiac diseases are strongly associated with aging and pose a major threat to survival. This study evaluated the cardioprotective effects of oral eugenol and its nano-emulsion (20 mg/kg) in a D-galactose-induced aging model (300 mg/kg, i.p.) in male and female rats over 12 weeks. Particle size and zeta potential analyses confirmed the successful development of a stable, well-dispersed nano-emulsion system. At the end of treatment, echocardiography, biochemical assays, histopathology, and immunohistochemistry were performed. Cardiac dysfunction and dilatation were more pronounced in males than females following D-galactose administration, as evidenced by left ventricular internal diameter in diastole (LVIDd) and left ventricular internal diameter in systole (LVIDs), and reduced ejection fraction (EF) and fractional shortening (FS). Both eugenol and its nano-emulsion preserved cardiac architecture and mitigated histopathological alterations, including myofibrillar distortion, necrosis, vascular remodeling, and fibrosis. Treatment significantly reduced oxidative stress and mitochondrial dysfunction by restoring GSH, lowering MDA, and modulating the PINK1/Mfn2 pathway. Furthermore, eugenol and its nano-emulsion attenuated cardiac inflammation, apoptosis, and fibrosis through downregulation of TGF-β1, MMP-9, TNF-α, and caspase-3 expression. In conclusion, eugenol nano-emulsion demonstrates promising anti-aging potential against cardiac complications by regulating mitochondrial dynamics, mitophagy, oxidative stress, inflammatory and apoptotic responses, and the SIRT1/TGF-β/MMP9 signaling pathway.

**Supplementary Information:**

The online version contains supplementary material available at 10.1007/s00210-026-05262-4.

## Background

Aging is a natural, progressive, inescapable physiological phenomenon that is associated with structural and functional deterioration in cells (Bhatiya et al. 2023). Cardiac tissue is vulnerable to changes related to aging. These physiological and structural alterations affect heart function. Transformation in the primary function of cardiomyocytes is attributed to age-related changes, which may be elicited by a wide range of endogenous and exogenous stimuli such as chronic inflammation, mitochondrial dysfunction, oxidative stress, cumulative telomere attrition, epigenetic adjustment, and impaired protease secretion (Peng et al. 2025). Cardiac dysfunction manifests as left ventricular dilatation, reduced left ventricular diastolic function, atrial fibrillation, left atrial dilation, myocardial fibrosis, and cardiac amyloidosis, and increased predisposition to chronic heart failure in aged individuals. Not only the cardiac tissue but also the vascular system is affected by age-related changes, leading to arterial stiffness, endothelial dysfunction, and malfunctioning angiogenesis (Fang et al. 2024).

Mitochondrial dysfunction participates intensively in cardiovascular aging-related disorders. Cardiac malfunction stems from massive reactive oxygen species (ROS) production and oxidative damage that results from accumulated uneliminated damaged mitochondria (Yang 2025). Interrupted mitochondrial dynamics and disrupted mitophagy provoked by aging result in the generation of free radicals, mitochondrial deterioration, and leakage of cytochrome C. Nevertheless, under normal conditions, these impaired mitochondria are removed through mitophagy, which is mediated by the PTEN-induced kinase 1 (PINK1) pathway (Strack 2020). Moreover, Mitofusin 2 (Mfn2) has been reported to have a chief role in the regulation of mitochondrial dynamics as it preserves mitochondrial quality and is involved in other important processes such as mitophagy (Chen et al. 2003; Chen and Dorn 2013).

Furthermore, the destructive cycle of ROS production and mitochondrial damage eventually leads to programmed cell death, which was revealed that minimal levels of apoptosis were enough to cause cardiomyocyte loss, leading to left ventricular dilatation and decreased ventricular function (Pereira et al. 2018).

Sirtuin1 (SIRT1) belongs to the sirtuin family, which comprises seven members: SIRT1-7. They are NAD + -dependent deacetylases and genetically stable across species. However, they control biological processes such as metabolism, autophagy, DNA restoration, as well as mitochondrial function maintenance, redox homeostasis, and senescence (Matsushima and Sadoshima 2015). On the other hand, decreasing mitochondrial biogenesis through activating the (SIRT1)/PINK-1 pathway in the heart improves cardiac dilatation (Guan et al. 2023). In addition, oxidative stress during aging leads to cardiac apoptosis (Chen et al. 2011).

Abnormal metabolic processes such as glucose oxidation promote excessive ROS production, which in turn results in increased regulation of transforming growth factor β (TGF-β) and extracellular matrix (ECM) remodeling, which contributes to the progression of cardiac fibrosis (Richter and Kietzmann 2016). Besides ECM production, fibroblasts also produce matrix metalloproteinase (MMPs), including MMP9 (Moore et al. 2012). MMP9 and TGF-β are somehow related to each other, as it was documented that MMP9 stimulates latent TGF-β, and other evidence assumed that TGF-β upregulates MMP9 through the ROS signaling pathway (Perng et al. 2011; Zhang et al. 2013). Cardiac aging has been accompanied by cardiac vascular remodeling, left ventricular dilatation, and fibrosis. Thus, cardiac fibrosis is considered a hallmark of the aging heart (Chen and Frangogiannis 2010).

The most commonly used method for mimicking the aging process is the D-galactose model in rodents. D-galactose is a reducing sugar that leads to ROS production, inflammation, disturbed cellular metabolism, cell injury, and ultimately cardiac dilatation, as well as structural and functional alterations in the cardiovascular system (Cheng et al. 2021).

Eugenol is a natural phenolic compound extracted from cinnamon oil, nutmeg oil, clove oil, and bay leaf oil, as well as other essential oils. Many studies have revealed its anti-inflammatory, antioxidant, and anti-hyperglycemic properties of eugenol and have presumed that eugenol attenuates the production of cytokines, chemokines, and other inflammatory mediators (Al-Trad et al. 2019). Additionally, Fen et al. (2018) validated the beneficial impact of eugenol against ischemia/reperfusion injury in the transplanted heart in rats through dampening inflammatory and apoptotic responses. Also, the cardioprotective impact of eugenol against hypertension and heart toxicity brought on by doxorubicin in rats has been documented (Soliman et al. 2024). These properties suggest that eugenol may be able to reduce the risk of cardiovascular diseases (CVDs) (Devi et al. 2024).

Despite eugenol having many beneficial properties, some of its characteristics as being water insoluble, highly volatile, and unstable when exposed to light, heat, oxygen, and humidity hinder the generation of pharmaceutical formulations and reduce their effectiveness. The nano-emulsion formula of bioactive compounds, especially lipophilic ones like eugenol, provides advantages in producing stable aqueous systems for targeted delivery, concentrating on enhancing factors such as degradation prevention, assuring physicochemical stability, increasing water solubility, optimizing bioavailability, and enhancing membrane permeability (Wilson et al. 2022). Hence, this current study investigates the possible potential impact of eugenol or its constructed nano-emulsion on cardiovascular disorders induced by D-galactose in male and female rats, focusing on gender-related differences.

## Materials and methods

### Animals

Seventy-two Wistar rats (36 males and 36 mature non-pregnant females) weighing 180 ± 20 g (3 months age) were purchased from Vacsera, Helwan, Egypt. They were kept for 2 weeks for acclimatization in plastic cages with sawdust bedding under proper environmental conditions (22 ± 5 °C, 50 ± 5% humidity, and a 12-h/12-h light/dark cycle) with water provided through dropper-tipped bottles and pellet chow available ad libitum (Department of Pathology, Faculty of Veterinary Medicine, Cairo University).

### Ethics

The protocol was approved by the Institutional Animal Care and Use Committee (IACUC) of Cairo University (approval number: Vet CU110520251121). By approving the experimental protocol, the committee ensured that it complied with the ARRIVE criteria and the guidelines outlined in the Guide for Care and Use of Laboratory Animals (NIH Publications No. 8023, revised 1978).

### Chemicals

D-galactose (C₆H₁₂O₆; molecular weight 180.16 g/mol; purity (HPLC) ≥ 98.0%) was purchased from Merck KGaA, Darmstadt, Germany (lot No. K47822258638). Eugenol (HPLC grade, 99% purity) and Tween 20 were obtained from Sigma-Aldrich, St. Louis, MD, USA; lot No: E51791-99.

### Nano formulation

Eugenol nano-emulsion was prepared using the low-energy emulsification method with Tween 20 as the non-ionic surfactant. The formulation consisted of 0.8% eugenol, 2.4% Tween 20 as surfactant, and 96.8% Millipore water (Aboelsoued et al. 2024). The components were mixed under magnetic stirring at 800 rpm for 15 min to ensure homogeneity, followed by ultrasonication for 11 min to reduce droplet size and enhance stability. The resulting nano-emulsion appeared as a transparent to slightly bluish dispersion, indicating successful formation of nanosized oil droplets.

The particle size and zeta potential of eugenol nano-emulsion were analyzed using NICOMP 380 ZLS, PSS, Santa Barbara, CA, USA. The particle size was measured according to dynamic light scattering with 700 times the laser beam scattering with eugenol nano-emulsion particles. Zeta potential was measured as particle charge through applied electrical current on alloy electrodes.

### Experimental design

A total of 72 rats (36 males and 36 females) were randomly distributed and equally divided into 12 groups (6 males and 6 females) each (*n* = 6); where the control group was kept untreated, group 2 received eugenol in olive oil at a dose of 20 mg/kg (0.5 mL/200 g rat bw) orally for 12 weeks (El-Far et al. 2022; Carvalho et al. 2025). Group 3 received eugenol nano-emulsion at a dose of 20 mg/kg orally for 12 weeks, while group4 (D-GAL, model group) was injected 300 mg/kg D-galactose dissolved in saline intraperitoneally for 12 weeks (Peng et al. 2025). Group 5 (D-GAL + eugenol) received 20 mg/kg (0.5 ml/200 g rat bw) of eugenol + 300 mg/kg D-galactose for 12 weeks, and group 6 (D-GAL + nano-eugenol) received 20 mg/kg eugenol nano-emulsion (0.5 mL/200 g rat bw) + 300 mg/kg D-galactose for 12 weeks (Sober et al. 1950).

#### Echocardiograph

In the last week of the experiment, three rats from each group were anaesthetized using IP injection of xylazine/ketamine mixture (Forman et al. 1997; Azar et al. 2014). Rats were placed in a supine position and transthoracic echocardiography was conducted using an Aloka F31 unit (Hitachi company, Japan) with a 13-MHz transducer. M-mode was recorded from the parasternal short axis view, and then measurements were averaged from three consecutive cycles. LV internal diameter (LVID), interventricular septal thickness (IVS), and LV posterior wall (LVW) were recorded at both end-systole and diastole, then systolic indices; fractional shortening (FS%) and ejection fraction (EF%) were calculated as previously formulated (Sahn et al. 1978).

#### Experimental endpoint and tissue collection

At the end of the experimental period, rats were humanely euthanized by cervical dislocation for tissue sample collection. Heart tissues were dissected; parts of the tissues were fixed in 10% formalin for histopathological examination and immunohistochemistry, while other parts were stored at − 80 °C for biochemical analysis interventions.

#### Biochemical analyses

##### Preparation for cardiac homogenate

Cardiac tissues were rinsed in ice-cold PBS (0.01 mol/L, pH 7.0–7.2) to eliminate excess blood and homogenized after being weighed using a polytron homogenizer. The resulting suspension was then sonicated with an ultrasonic cell disruptor. Then, the homogenates were centrifuged for 5 min at 5000 × *g*. The supernatant was then carefully isolated and immediately used for the assessment of oxidative stress biomarkers, while the remaining aliquots were stored at ≤  − 20 °C for further biochemical analyses. The protein content of the tissue was determined following the Bradford method using a protein estimation kit supplied by Genei, Bangalore.

##### Determination of oxidative stress and antioxidant markers

As instructed by the manufacturer, “Biodiagnostic Company, Dokki, Giza, Egypt” kits were used to assay the levels of GSH (reduced glutathione) content and levels of MDA (Malondialdehyde) in the heart homogenate.

##### Enzyme-linked immunosorbent assay (ELISA) on tissue homogenate

Rat SIRT1 was assessed using an ELISA Kit (Cat No: BSKR65911, Bioss Antibodies, Boston, USA), rat TGF-β1 was assessed using an ELISA Kit (Cat No.: ER1378, FineTest, Wuhan, Hubei, China), and MMP9 was evaluated using a rat ELISA kit (Cat No.: SEA553Ra, Houston, USA). Rat ELISA Kit (Cat No.: SLD1769Ra, Hangzhou City, Zhejiang Province, China) was used to assess PINK-1, and rat Mfn2 (Mitofusin 2) was assessed using the ELISA Kit (Cat No.: ELK0352, Denver, USA).

### Histopathological examination

Cardiac tissues were preserved in 10% neutral buffered formalin till complete fixation, then processed in ascending concentration of ethanol and xylene, embedded in paraffin wax blocks, sectioned (5-μm thick), and stained with hematoxylin and eosin (Bancroft and Gamble 2007). Slides were examined and photographed using an Olympus BX50 light microscope (Japan). Furthermore, some cardiac tissue sections were stained with Picrosirius red to evaluate cardiac fibrosis and examined by bright-field and polarized microscopy.

### Immunohistochemical analysis

Immunohistochemistry of caspase-3 and TNF-α was performed in paraffin-embedded tissue sections after deparaffinization, rehydration, and antigen retrieval using citrate buffer PH 6, anti-caspase-3 antibody (YPA1086; 1:100 dilution rate, Biospes, China) and anti-TNF alpha antibody (sc-52746; 1:100 dilution rate, Santa Cruz, USA) were applied to the tissue overnight. Hydrogen peroxidase blocker (Thermo Scientific, USA) was used, followed by the application of biotinylated secondary antibody and avidin horse radish peroxidase according to the manufacturer’s protocol (Cat. No. 0001TOA12, Biospes, China). DAB chromogen was used for color development. Five heart sections per group were analyzed to investigate the immunoreaction of caspase-3 and TNF-α (Mowaad et al. 2025). Area percent of positively stained cells (%) was evaluated using ImageJ 1.52 p software (Wayne Rasband, National Institutes of Health, USA) in five images per rat at × 400 magnification power.

### Statistical analysis

All data generated in this study were analyzed using GraphPad Prism software (version 9). Data normality was verified using the Shapiro–Wilk test, confirming normal distribution at *p* > 0.05. Statistical comparisons were performed using two-way analysis of variance (two-way ANOVA), followed by Tukey’s post hoc multiple comparison test to determine intergroup differences. All data are presented as mean ± standard error (SE), and a *p*-value < 0.05 was considered statistically significant.

## Results

### Characterization of nano-eugenol

**Fig. 1:**
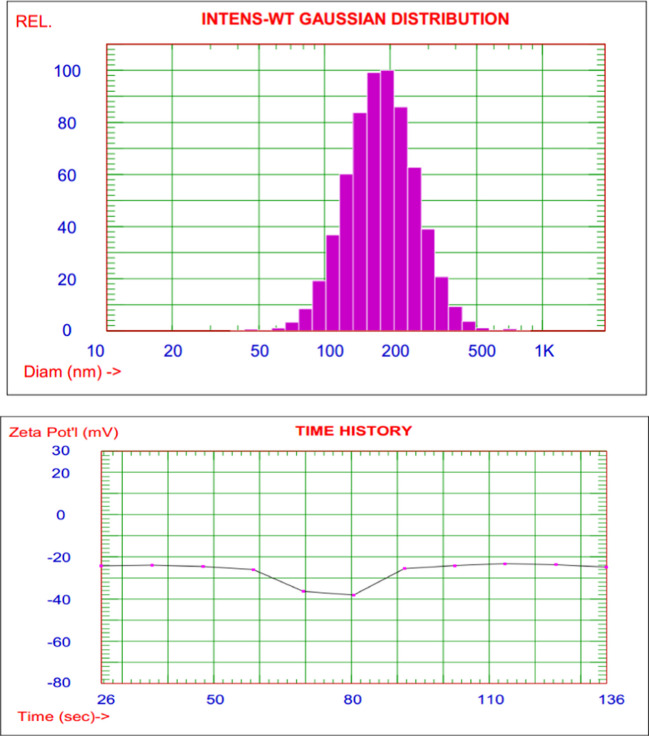
Particle size distribution and zeta potential of eugenol nano-emulsion

The particle size distribution results indicated that the eugenol nano-emulsion exhibited a Gaussian distribution with droplet diameters predominantly in the range of 100–250 nm, with the peak intensity centered around 165 nm, as shown in Fig. 1. This narrow distribution reflects good uniformity of droplet size and suggests effective emulsification and stabilization by Tween 20. The nanoscale droplet size is favorable for enhancing surface area, bioavailability, and stability of the encapsulated eugenol. Since no significant presence of larger aggregates (> 500 nm) was observed, the formulation can be considered stable with minimal risk of phase separation. Overall, the particle size analysis confirms the successful formation of a well-dispersed nano-emulsion system.

The zeta potential results revealed values ranging between − 20 and − 38 mV throughout the measured time interval, as presented in Fig. 1. A noticeable dip toward − 38 mV was observed midway, followed by a recovery closer to − 20 mV. These values indicate that the nano-emulsion droplets possess a good, stable negative surface charge. The eugenol nano-emulsion prepared with Tween 20 demonstrates acceptable colloidal stability as a long-term stable nano-emulsion.

### Impact of eugenol and its nano-emulsion on heart function parameters

**Fig. 2:**
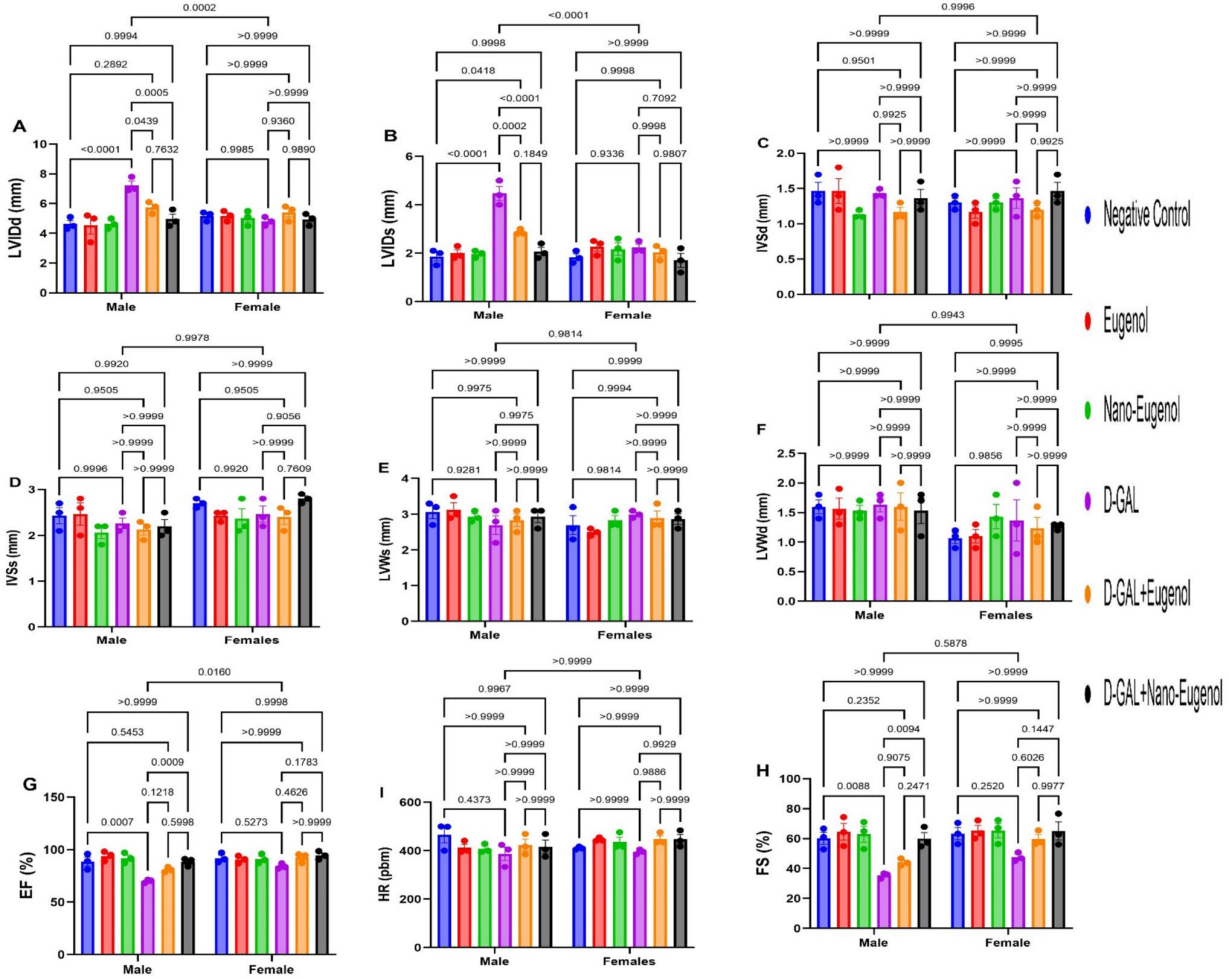
Impact of eugenol and its nano-emulsion on heart function parameters in male rats treated groups. D-GAL, D-galactose. IVSd**,** interventricular septal thickness in diastole. LVIDd, left ventricular internal diameter in diastole. LVWd, left ventricular posterior wall thickness in diastole. IVSs, interventricular septal thickness in systole. LVIDs, left ventricular internal diameter in systole. LVWs, left ventricular posterior wall thickness in systole

The ECHO measurements for all groups are statistically analyzed and summarized in Fig. 2 and Fig. 1S (Supplementary file). In male rats, there was no significant difference between the baseline HR, IVS, and LVW and other groups in both systole and diastole. The LVIDd was statistically higher in the D-galactose and D-galactose + eugenol-treated group than in the other groups. The D-galactose-injected group had the highest significant value for LVIDs, while there was no significant difference between D-galactose + eugenol- and D-galactose + nano-eugenol-treated groups. However, LVIDs showed no difference between the baseline and eugenol- and nano-eugenol-treated groups. The systolic function, as indicated with the FS% and EF%, was significantly impaired in the D-galactose-injected group; however, it was not statistically significant from the D-galactose + eugenol-treated group. In female rats, there was no difference between the baseline ECHO measurements and other groups. Two-way ANOVA analyses demonstrated significant differences in LVIDd (*p* = 0.0002), LVIDs (*p* < 0.0001), and.

EF% (*p* < 0.016) between male and female rats in D-galactose-treated groups.

### Impact of eugenol and its nano-emulsion on cardiac SIRT1/ROS pathway

**Fig. 3:**
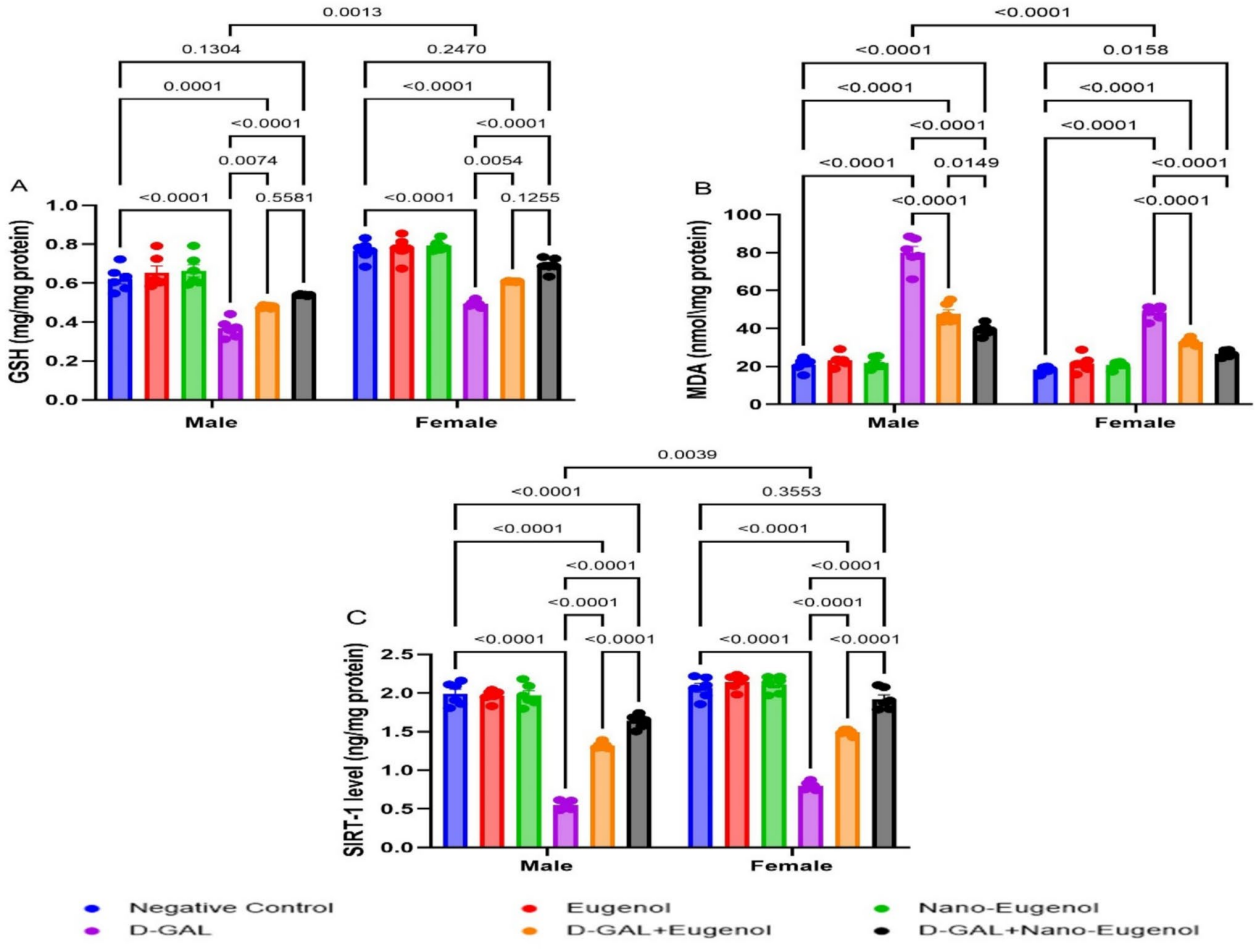
Impact of eugenol and its nano-emulsion on changes of cardiac SIRT1/ROS pathway; GSH (**A**), MDA (**B**), and SIRT1 (**C**) in D-galactose-induced aging in male and female rats. Data were expressed as mean ± SE. Two-way ANOVA followed by Tukey’s post hoc multiple comparison test to determine intergroup differences

GSH content declined significantly in both male (41.15%) and female (35.68%) rats in the D-galactose-injected groups, relative to control groups. In contrast, D-galactose administration elevated MDA level in male and female rats by 3.8-fold and 2.64-fold, respectively. On the other hand, GSH level was improved by 30.61% and 23.31% and MDA content was diminished by 40.22% and 31.94% in male and female rats treated with D-galactose + eugenol. Nano-eugenol significantly raised the GSH levels by 47.19% and 40.28%, respectively, as well as decreased the MDA contents by 50.79% and 44.82% in D-galactose-injected male and female rats, respectively. It should be noted that nano-eugenol showed better antioxidant capability than its native form (Fig. 3A and B).

SIRT1 was significantly decreased in the heart tissue by 71.15% and 64.29% in male and female rats injected with D-galactose, respectively, in relative to their corresponding control rats. On the other hand, eugenol and its nano-emulsion markedly restored the cardiac SIRT1 level, reaching 138.6% and 215%, respectively, in male animals, and to 102.1% and 160.24%, respectively, in female-treated animals, compared to their corresponding control groups (Fig. 3C). Two-way ANOVA revealed no significant differences between male and female rats in the control groups. However, under oxidative stress conditions induced by D-galactose, a significant main effect of sex was observed for cardiac GSH (*p* = 0.0013), MDA (*p* < 0.0001), and SIRT-1 (*p* = 0.0039). Specifically, GSH and SIRT-1 levels were more markedly decreased in males by 25.73% and 25.06%, respectively, compared to females, while MDA levels were significantly higher in males by 65.15%, indicating that males are more vulnerable to oxidative stress than females.

### Impact of eugenol and its nano-emulsion on cardiac TGF-β/MMP-9 pathway

**Fig. 4:**
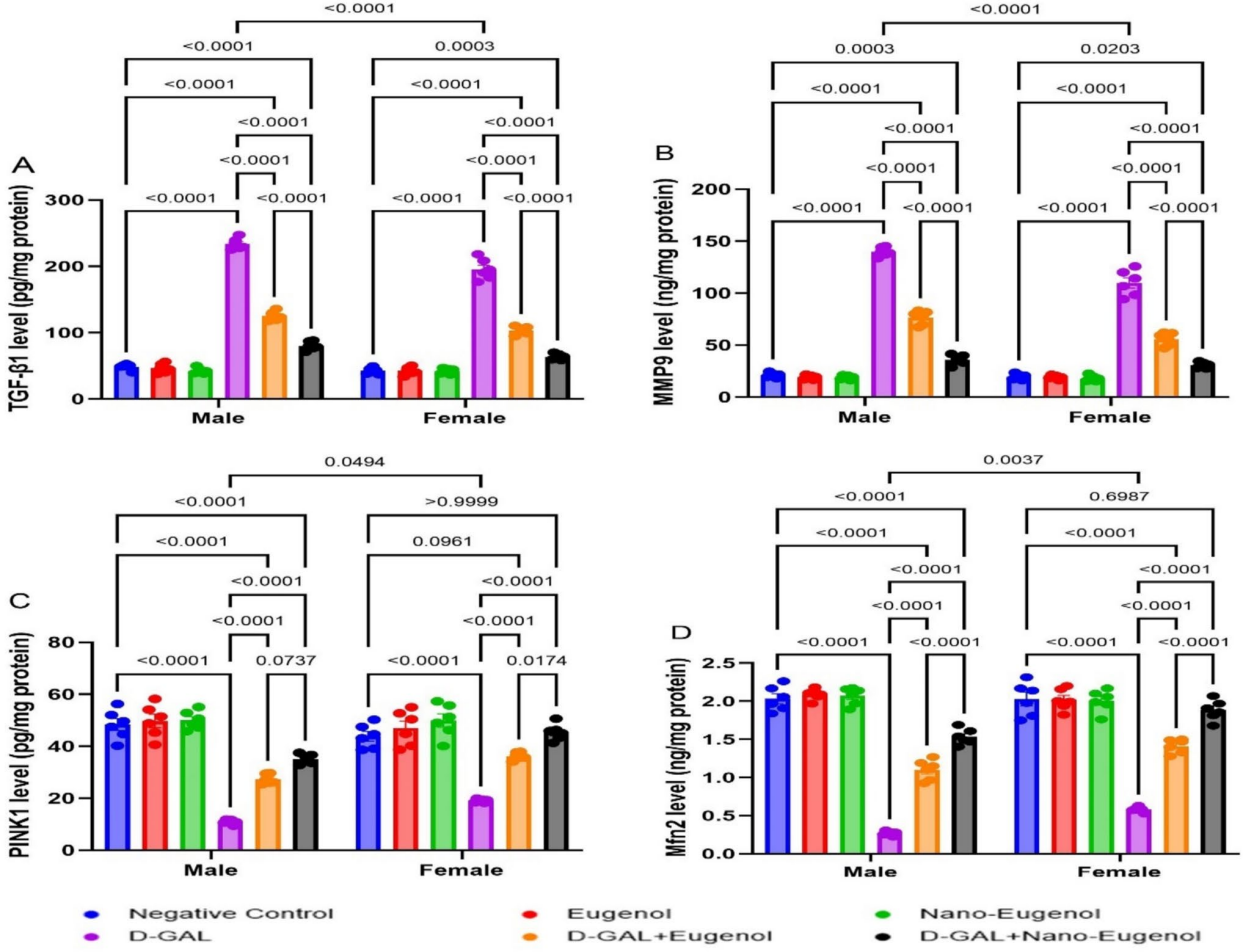
Impact of eugenol and its nano-emulsion on changes of cardiac profibrotic markers; TGF-β (**A**) and MMP9 (**B**) levels in D-galactose-induced aging in male and female rats. Impact of eugenol and its nano-emulsion on changes of cardiac mitophagy and mitochondrial dynamics markers; PINK1 (**C**) and Mfn2 (**D**) levels in D-galactose-induced aging in male and female rats. Data were expressed as mean ± SE. Two-way ANOVA followed by Tukey’s post hoc multiple comparison test to determine intergroup differences

When compared to the negative control rats, a marked boost in the cardiac TGF-β (4.8-fold and 4.2-fold) and MMP9 (6.63-fold and 5.57-fold) content has been detected in both male and female aged model rats, respectively. Conversely, oral administration of eugenol significantly decreased the levels of cardiac TGF-β by 46.23% and 47.07% and MMP9 by 45.22% and 49.09% in D-galactose-injected male and female rats, respectively. Additionally, in D-galactose + nano-eugenol-treated male and female rats, marked improvement in the levels of TGF-β reaching 65.68% and 67.26%, respectively, as well as MMP9 (74.33% and 72.08%), respectively, has been demonstrated (Fig. 4A and B). Two-way ANOVA demonstrated no significant differences between male and female rats in the control groups. However, under pathologic conditions induced by D-galactose, significant differences were observed in the level of cardiac MMP-9 (*p* < 0.0001), and TGF-β1 (*p* < 0.0001) between male and female rats of D-galactose-treated groups, highlighting the main effect of sex factor (Fig. 4). Explicitly, MMP 9 and TGF-β1 levels were more markedly increased in males by 27.22% and 19.84%, respectively, compared to females, indicating that males are more susceptible to cardiac fibrosis by aging than females (Fig. 4).

### Impact of eugenol and its nano-emulsion on cardiac PINK1/Mfn2 pathway

As shown in (Fig. 4), a significant decrease in PINK1 by 73.24% and 61.46% and Mfn2 by 85.98% and 78.8% has been reported in the heart tissue of D-galactose-injected male and female rats, respectively, verifying the disrupted mitochondrial dynamics and mitophagy impairment, compared to the negative control ones. On the other hand, eugenol and its nano-emulsion treatment to male and female rats injected with D-galactose for 3 months resulted in a marked rise in the cardiac PINK1 (126.45% and 96.74%) and Mfn2 (321.62% and 247.34%) in eugenol-treated groups while for nano-eugenol-treated groups, the improvement in PINK1 and Mfn2 reached 222.67% and 217.85%, and 552.02% and 373.1%, respectively, with respect to the model groups. Nano-eugenol showed a better improvement effect than its native form concerning the mitochondrial dynamics and mitophagy mediators in the treated groups. The cardiac PINK1/Mfn2 pathway downregulation was more prominent in the male model group than the female ones, as illustrated in (Fig. 4C and D). Two-way ANOVA verified that no significant differences were detected between male and female rats in the control groups. However, under pathological conditions induced by D-galactose, significant sex-related differences were observed in cardiac Mfn2 (*p* = 0.0037) and PINK1 (*p* = 0.049) levels, indicating a significant main effect of the sex factor. Notably, Mfn2 and PINK1 levels were more markedly reduced in males by 33.65% and 26.03%, respectively, compared to females, suggesting that males are more susceptible to aging-associated mitochondrial dysfunction than females.

### Histopathological findings

**Fig. 5:**
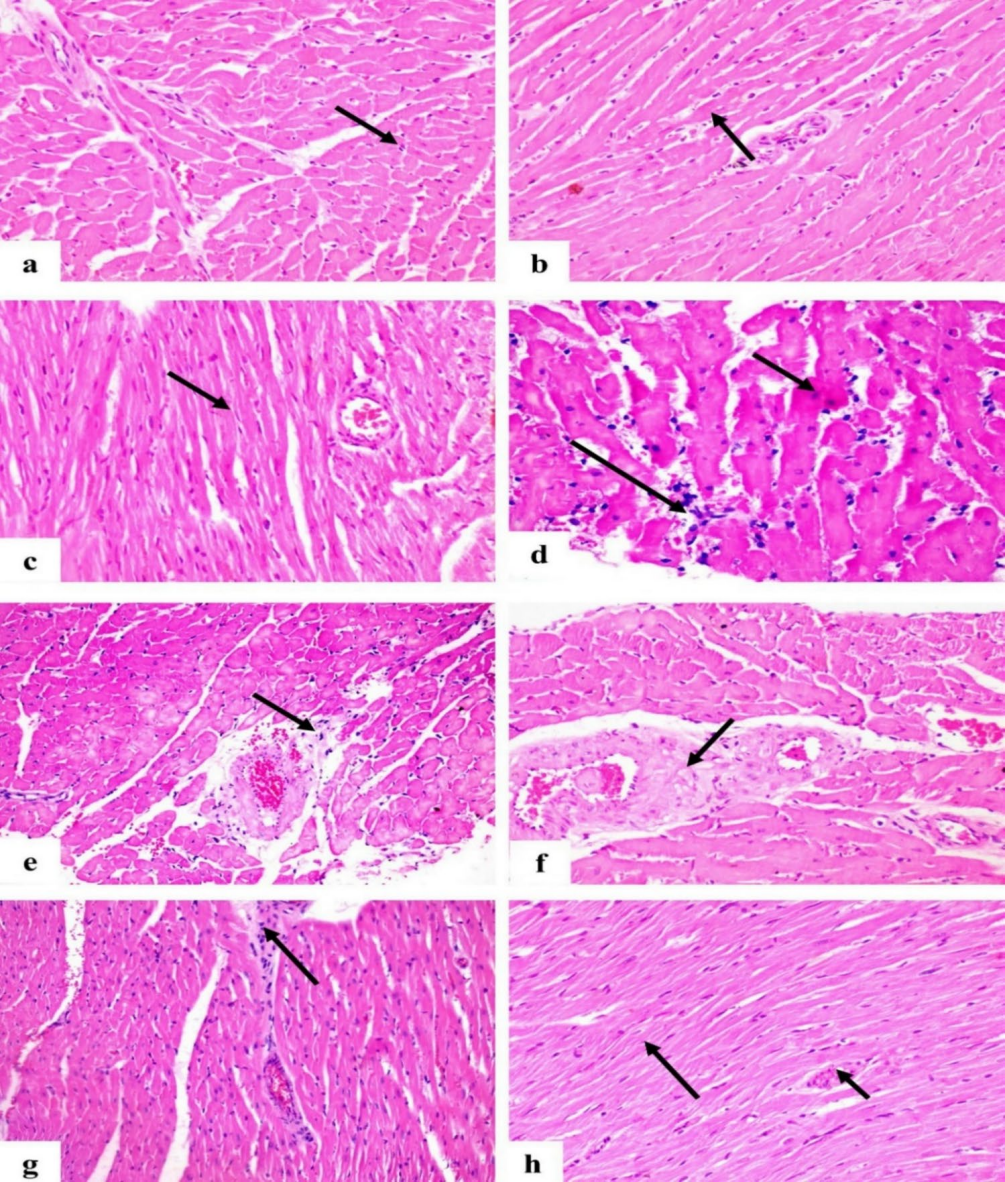
Photomicrographs of the male rats heart in different groups. Normal histological structure in **a** negative control group, **b** eugenol group, and **c** eugenol nano-emulsion group (arrows). **d** Zenker’s necrosis in myocytes (short arrow), **e** perivascular fibrosis (arrow), and **f** vascular remodeling with endothelial vacuolation and hypertrophy of tunica media (arrow) in the D-GAL-treated group. **g** moderate vascular remodeling (arrows) in the D-GAL + eugenol-treated group **h**, nearly normal vascular thickness (short arrow) and intact myocytes (long arrows) in the D-GAL + nano-eugenol group (hematoxylin and eosin stain × 200)

**Fig. 6:**
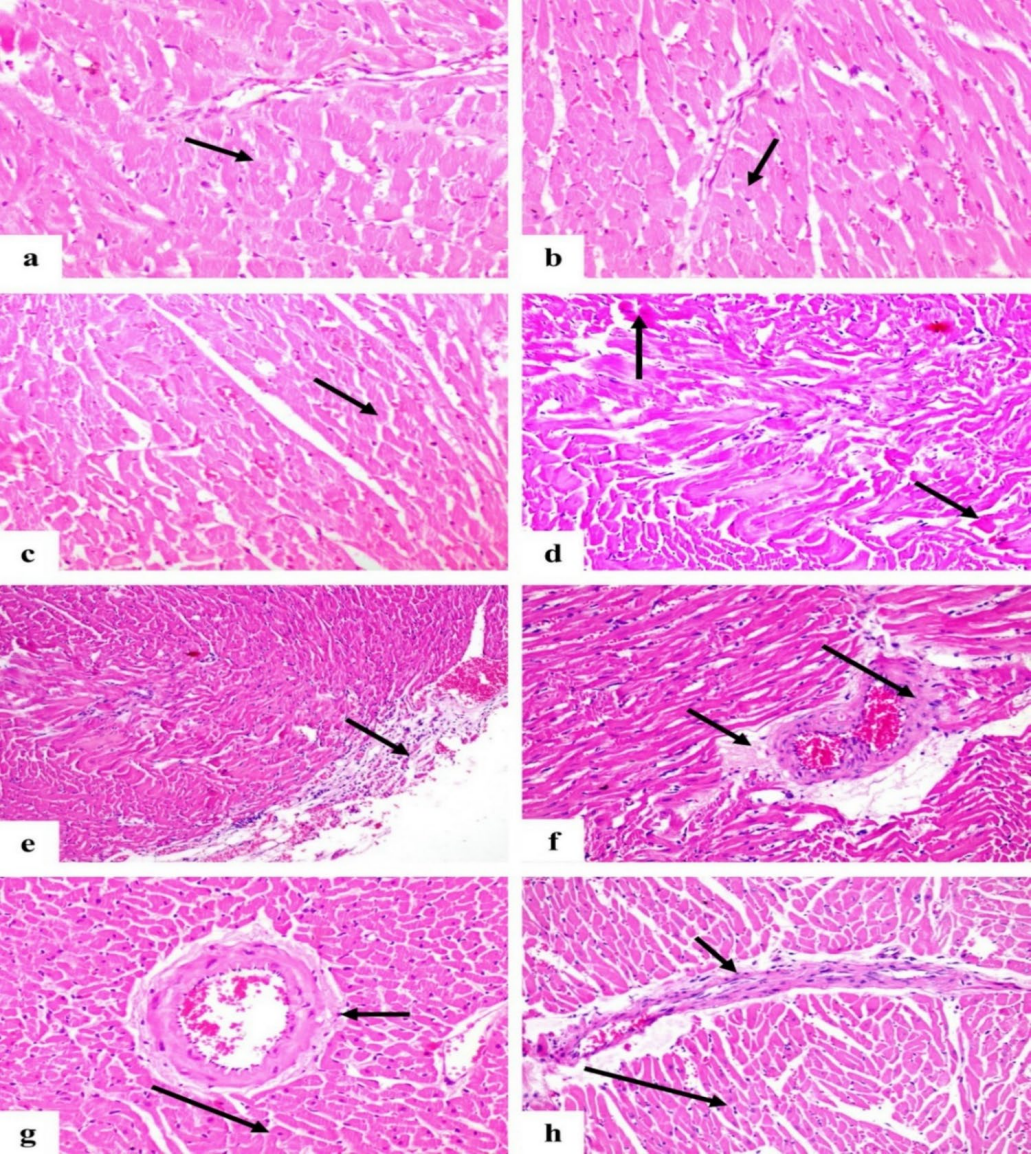
Photomicrographs of the female rats heart in different groups: **a** normal histological structure of heart in negative control group, **b** eugenol group, and **c** nano-eugenol group (arrows). **d** Deeply eosinophilic myocytes suffering from Zenker’s necrosis with loss of cross-striation (arrow) in the D-GAL-treated group. **e** Pericardium infiltrated with mononuclear inflammatory cells and slight hemorrhage (arrow) in the D-GAL-treated group. **f** Vascular remodeling (long arrow) and perivascular fibrosis and edema (short arrow) in D-GAL-treated groups **g**, moderate vascular thickening (short arrow) and intact myocytes (long arrow) in D-GAL + eugenol-treated group. **h** Normal vascular thickness (short arrow) and intact myocytes (long arrow) in D-GAL + nano-eugenol group (hematoxylin and eosin stain × 200)

Microscopy of the heart revealed normal histological structure in the control group, eugenol group, and nano-eugenol group (Figs. 5 and 6a, b, c), whereas in D-galactose-injected male and female rats, there were severe histological alterations as distorted and disarranged myocardial bundles, increased interstitial spaces, and Zenker’s necrosis of cardiac myocytes which appeared more eosinophilic with loss of sarcoplasm and cross striations (Figs. 5d and 6d). In addition, mononuclear inflammatory cell infiltration with moderate hemorrhage was observed in the pericardium (Fig. 6e). Furthermore, vascular remodeling was observed in cardiac blood vessels with congestion, vacuolated endothelial cells, thickening and hypertrophy of tunica media, and perivascular fibrosis and edema (Figs. 5f and 6f). On the other hand, D-galactose + eugenol showed maintained cardiac architecture with moderate vascular thickening and perivascular fibrosis (Figs. 5g and 6g). Nevertheless, the D-galactose + nano-eugenol group revealed a superior amelioration in cardiac tissue as the cardiomyocytes and vascular thickening were normal with no perivascular fibrosis, as shown in (Figs. 5h and 6h).

#### Assessment of collagen deposition using Picrosirius red stain

**Fig. 7:**
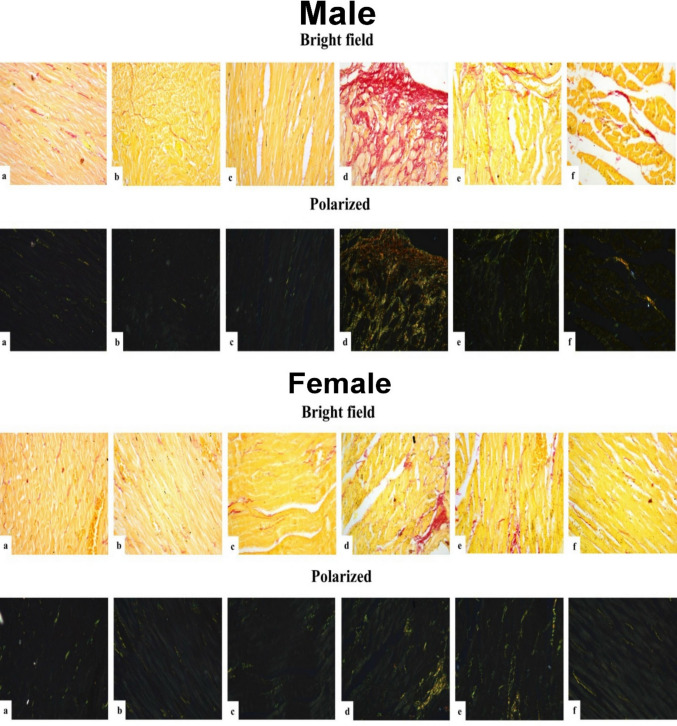
Photomicrographs of collagen stained with Picrosirius red stain in male and female rats’ heart tissue. Male: weak fibrosis in (**a**) negative control group, (**b**) eugenol group, and (**c**) eugenol nano-emulsion group, (**d**) severe interstitial fibrosis in D-galactose treated group, (**e**) moderate interstitial fibrosis in D-galactose + eugenol-treated group, (**f**) showing mild interstitial fibrosis in D-galactose + nano-eugenol. Female: weak fibrosis in (a) negative control group, (b) eugenol group, and (c) eugenol nano-emulsion group, (d) interstitial fibrosis in D-galactose treated group, (e) moderate interstitial fibrosis in D-galactose + eugenol treated group. (f) Mild interstitial fibrosis in D-galactose + nano-eugenol (picrosirius red stain × 200)

Normal feeble collagen fibers were observed in cardiac tissue of male and female rats in control groups (Fig. 7a, b, c). Conversely, severe diffuse reactive fibrosis was observed in D-galactose-injected male rats (Fig. 7d) compared to the control group. Collagen deposition was higher in D-galactose-injected female rats compared to their relative control groups, but much less than that of the induced aging male group (Fig. 7d). The treatment with eugenol and eugenol nano-emulsion markedly reduced fibrosis in male and female induced aging rats, as displayed in (Fig. 7e, f) in their relative order. The percentage of collagen in different groups is presented in (Fig. 9C). Two-way ANOVA revealed a significant difference in the area % of collagen deposition (*p* < 0.0001) between male and female rats of D-galactose-treated groups, validating the main effect of sex factor. Obviously, no significant differences were found between the male and female control groups.

### Immunohistochemical findings of caspase-3 and TNF-α in heart tissue

**Fig. 8:**
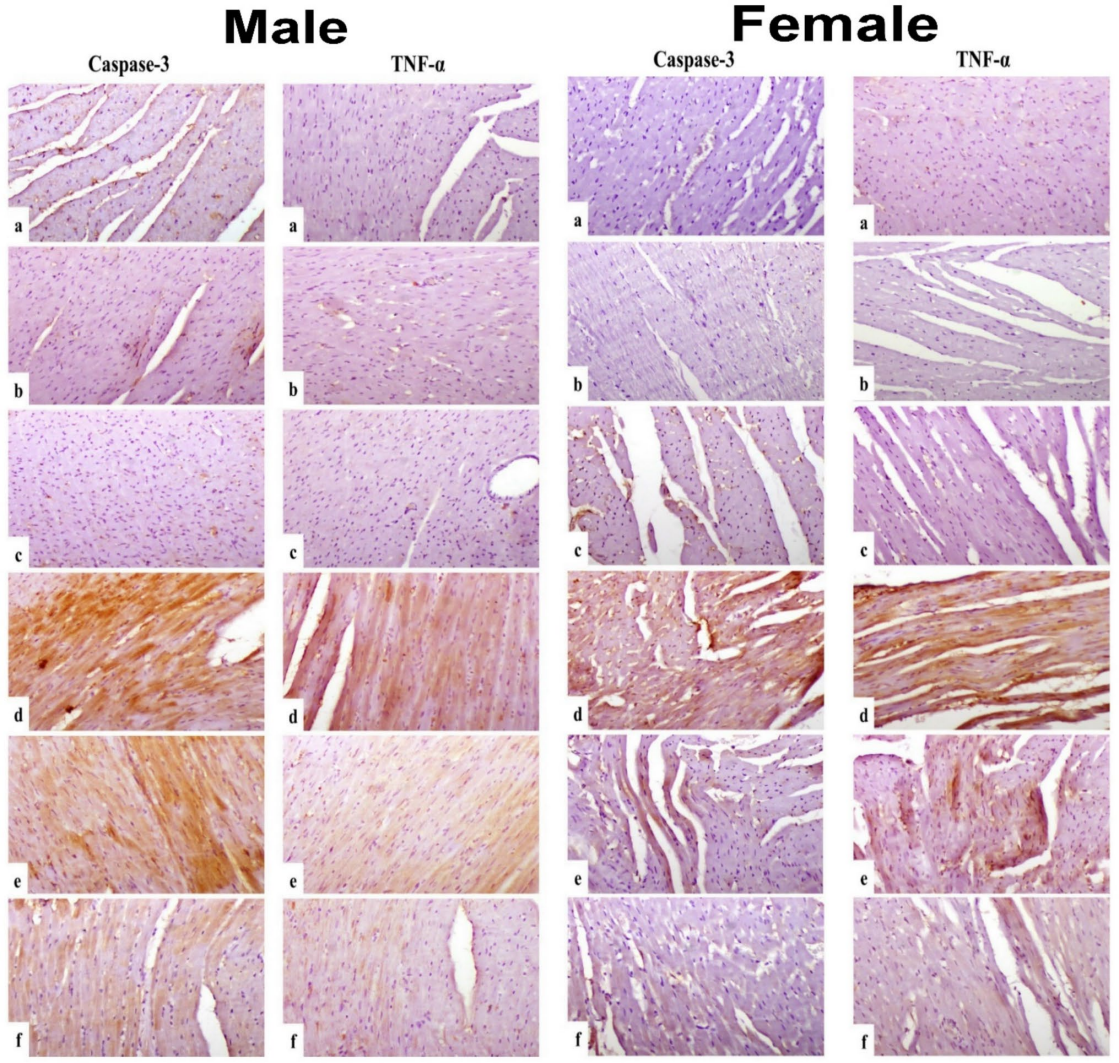
Photomicrographs of caspase-3 and TNF-α immunoexpression in male and female rat heart. Male: weak expression of both caspase-3 and TNF-α (**a**) negative control group, (**b**) eugenol group, (**c**) eugenol nano-emulsion group, (**d**) strong expression of caspase-3 and TNF-α in D-galactose-treated group, (**e**) moderate expression of caspase-3 and TNF-α in D-galactose + eugenol-treated group, (**f**) mild to moderate caspase-3 and TNF-α expression in D-galactose + nano-eugenol. Female: weak expression of both caspase-3 and TNF-α (a) negative control group, (b) eugenol group, (c) eugenol nano-emulsion group, (d) strong expression of caspase-3 and TNF-α in D-galactose-treated group, (e) moderate expression of caspase-3 and TNF-α in D-galactose + eugenol-treated group, (f) mild caspase-3 and TNF-α expression in D-galactose + nano-eugenol (immunoperoxidase and hematoxylin counterstain × 200)

**Fig. 9:**
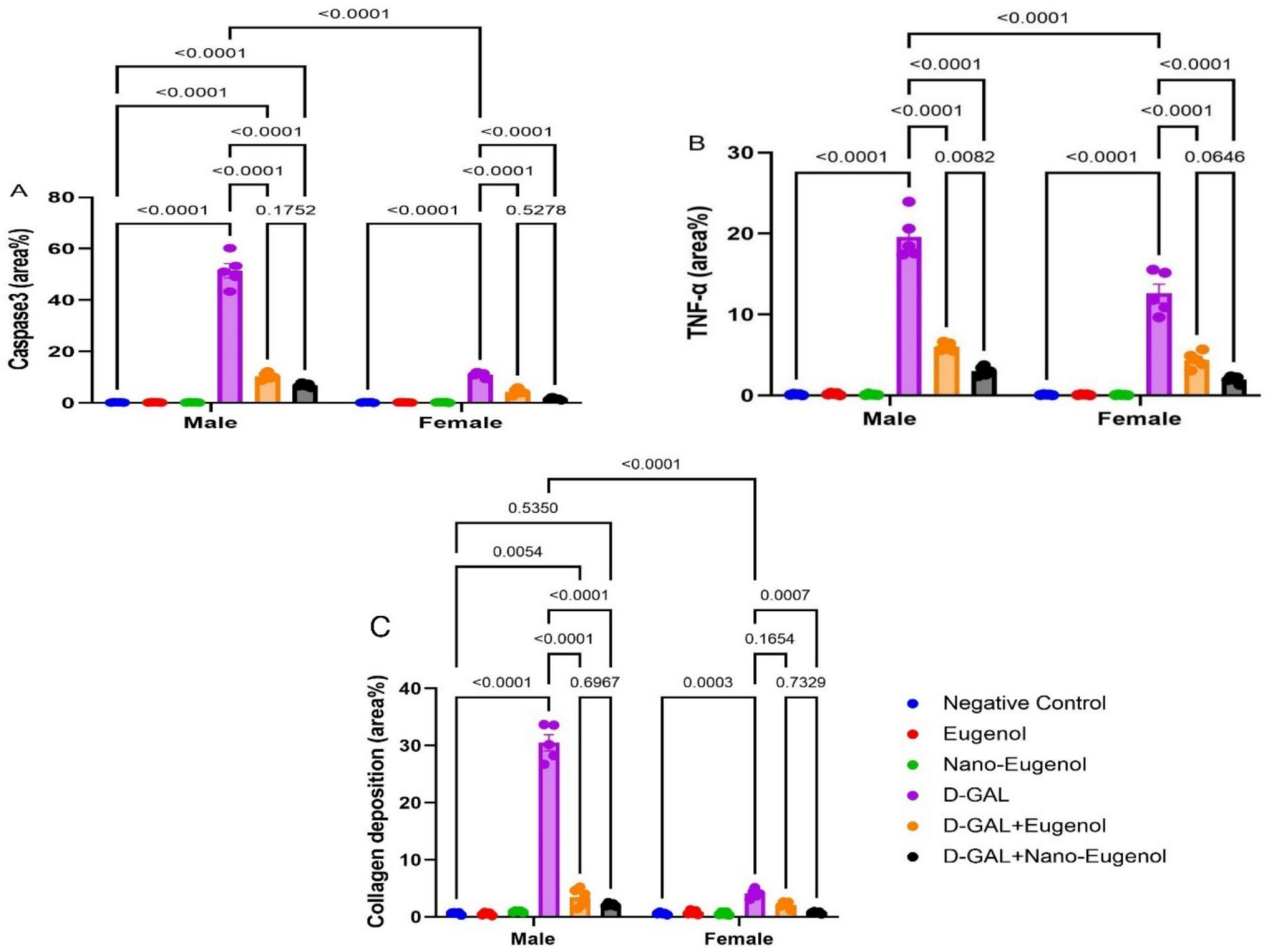
Area% of immune expression of Caspase-3 (**A**), TNF-α (**B**), and (**C**) collagen deposition in male and female groups. Data were expressed as mean ± SE. Two-way ANOVA followed by Tukey’s post hoc multiple comparison test to determine intergroup differences

Strong immunoreactivity of both caspase-3 and TNF-α was observed in D-galactose-treated male and female rats compared to their respective control groups (Fig. 8d), with a robust expression in male rats than in the female ones. Moderate expression of caspase-3 and TNF-α was noted in male rats of the D-galactose + eugenol- and D-galactose + nano-eugenol-treated groups, with significant improvement relative to the D-galactose-injected group, while a mild expression was observed in female rats compared to D-galactose-injected ones. The area% of caspase-3 and TNF-α immunoexpression in cardiac tissue is presented in (Fig. 9A, B). Two-way ANOVA revealed significant differences in the area% of caspase-3 (*p* < 0.0001) and TNF-α (*p* < 0.0001) between male and female rats of D-galactose-treated groups, validating the effect of the sex factor. Additionally, no significant differences were recorded between the male and female control groups.

## Discussion

Aging is recognized as the most significant risk factor for the development of CVD, which is the leading cause of death. With the aim of attenuating cardiovascular age-related disease, many studies have been established to mimic the process of natural aging and develop impactful, cost-effective anti-aging strategies. D-galactose-induced premature aging models exhibit similar phenotypes of cardiac changes that mimic natural aging in rodents (Peng et al. 2025).

This study provides new perspectives compared to previous reports on cardiac fibrosis. Unlike models based on pressure overload or chemical injury, we utilized a D-galactose-induced aging model to better reflect age-related oxidative stress and mitochondrial dysfunction. Moreover, by including both male and female rats, we revealed sex-related differences in susceptibility to cardiac dysfunction, an aspect often underexplored in earlier studies. Finally, our direct comparison between eugenol and its nano-emulsion formulation highlights the potential advantages of nano-delivery systems in enhancing cardioprotective efficacy. Together, these features distinguish our work from prior studies and contribute to a broader understanding of the mechanisms underlying age-related cardiac fibrosis.

Eugenol, a naturally occurring phenolic compound, has been recognized as a potent anti-inflammatory, antioxidant, and anti-fibrotic agent in both preclinical and clinical investigations (Damasceno et al. 2024). Nanotechnology of such phenolic compounds as eugenol represents a promising approach to overcome the poor oral absorption limitations and supports strategies to enable its practical application (El-Abd et al. 2025). In this context, the potential role of eugenol and its nano-emulsion has been investigated against D-galactose-triggered cardiac alteration in male and female Wistar rats for 12 weeks.

Cardiac tissue is vulnerable to changes related to aging. The echocardiography in the current study revealed that the cardiac function of male and female rats injected with D-galactose had declined, especially in males. This cardiac dysfunction was associated with increased LVIDd and LVIDs and reduced EF% and FS%, indicating cardiac dilation (Peng et al. 2025). Many pathological age-related histopathological vascular and cardiac alterations may contribute to functional decline, inducing many changes, such as apoptosis and fibrosis, that later lead to cardiovascular remodeling and hinder heart function. Vascular stiffening and thickening, loss of myocytes due to necrosis and apoptosis, and left ventricular thickening and enlargement were recorded with aging (Anversa et al. 1990; Lakatta and Levy 2003). This aligns with the results of histopathological examination in our study, as the heart tissue of D-GAL-injected rats showed vascular remodeling, inflammatory cells infiltration, Zenker’s necrosis, vacuolated endothelial cells, thickening and hypertrophy of tunica media, and perivascular and interstitial fibrosis and edema, which conforms with a previous study (Cheng et al. 2021). The mechanism investigated in the current study may clarify the structural and functional deterioration of the heart induced by aging.

Oxidative stress contributes to the occurrence of cardiac dilatation (D’Oria et al. 2020) and acts as a trigger that contributes to many destructive pathways. According to many studies, D-GAL triggers oxidative stress in the heart by suppressing many antioxidant enzymes and producing byproducts that are used as markers for oxidative damage (Shahidi et al. 2024; Mohamed et al. 2025). Our study showed that D-GAL-injected male and female rats had decreased levels of GSH, which is an essential non-enzymatic antioxidant in mammalian cells (Averill-Bates 2023) and is reported to decrease with age (Kasapoglu and Özben 2001; Kaplán et al. 2019) along with marked elevation in MDA, a byproduct of cell membrane polyunsaturated fatty acid peroxidation known as an indicator of oxidative stress when compared to control groups. Several investigations have been conducted to explore agents that could attenuate aging by upregulating SIRT1 (Matsushima and Sadoshima 2015). However, its expression seems to be impacted by accumulated ROS through post-translational modifications (Hwang et al. 2013). Similarly, in our study, it decreased in the heart of the D-GAL groups. In many experimental cases, eugenol opposed cardiotoxicity by elevating antioxidant enzymes such as SOD, CAT, and GSH (Mnafgui et al. 2015; Kumar et al. 2024). In the same context, our results revealed that eugenol and its nano-emulsion preserved the antioxidant/oxidant balance through increasing GSH production and diminishing lipid peroxidation processes in the heart tissue accompanied by decreased MDA liberation in addition to elevating SIRT1 (Liu et al. 2025).

A vicious cycle of ROS may result from the age-related mitochondrial dysfunction (Martín-Fernández and Gredilla 2016). It is documented that mitochondrial dysfunction contributes to various cardiomyopathies (Nargesi et al. 2019) and may trigger vascular smooth muscle cells proliferation leading to vascular stiffness and remodeling (Ye et al. 2021; Lu et al. 2021). Mitochondrial dynamics is a term that defines mitochondrial processes within the cell, which include fission, fusion, and movement (Fenton et al. 2021). Mitochondrial DNAis maintained by fusion and fission (Sabouny and Shutt 2021). Mfn2 not only maintains mitochondrial quality through fusion and fission but also is implicated in other crucial processes such as mitophagy, through acting as a receptor for PINK1/Parkin-mediated mitophagy (Chen and Dorn 2013). The PINK1/Parkin-mediated mitophagy controls the fate, survival, or death of cells by eliminating damaged mitochondria, thus strongly contributing to the pathogenesis of various CVDs (Jin et al. 2010). Interrupted mitochondrial function and dynamics was caused by ROS and reported to occur with aging, leading to further ROS production (Cui et al. 2012; Guo et al. 2023). Our aging models exhibited a significant downregulation of cardiac PINK1 and Mfn2 levels in both male and female rats treated with D-GAL leading to further oxidative damage due to accumulated ROS and uneliminated damaged mitochondria. However, eugenol and nano-eugenol alleviated mitophagy and mitochondrial function as groups treated with eugenol and nano-eugenol showed increased levels of both Mfn2 and PINK1.

The destructive cycle of ROS production and mitochondrial damage, along with decreased SIRT1, eventually leads to programmed cell death, since a minimal level of apoptosis was enough to cause cardiomyocyte loss, leading to left ventricular dilatation (Pereira et al. 2018). Likewise, immunohistochemical investigation in our study revealed high expression of caspase-3 in the heart tissue of D-GAL injected rats, along with high expression of tumor necrosis factor (TNF-α), a pro-inflammatory cytokine released in response to oxidative byproducts. Subsequently, this local inflammatory reaction leads further to the generation of free radicals, eventually leading to myocytes stretching, interstitial fibrosis, and cardiac dilatation (McGarry et al. 2018; Palomer et al. 2020). Remarkably, eugenol and nano-eugenol markedly ameliorated inflammation and apoptosis, which is consistent with previous studies reporting the anti-inflammatory and anti-apoptotic activity of eugenol in doxorubicin-induced cardiotoxicity and Cd-induced cardiac inflammation and dyslipidemia in rats (Kumar and Sharma 2024a, b).

Vascular remodeling and interstitial fibrosis can be attributed to the activation of TGF-β and MMP-9. TGF-β can be activated through various pathways, as it is thought to be upregulated by SIRT1 downregulation (Segura et al. 2014; Matsushima and Sadoshima 2015) and elevated ROS (Liu and Desai 2015). Evidence indicated that TGF-β reduces antioxidants, including GSH and other antioxidant enzymes, resulting in a redox homeostasis defect; such imbalance triggers TGF-β fibrotic action (Liu and Gaston Pravia 2010). Fibroblasts also produce matrix metalloproteinase (MMPs), including MMP9, which participates in cardiac remodeling in aged animals and increases with aging (Chiao et al. 2011; Moore et al. 2012). MMP9 and TGF-β are somehow related to each other, as MMP9 stimulates latent TGF-β, and other evidence suggests that TGF-β upregulates MMP9 through the ROS signaling pathway (Perng et al. 2011; Zhang et al. 2013). Interestingly, it was assumed that the age-associated upregulation of TGF-β had been ameliorated by MMP-9 downregulation (Chiao et al. 2012). Likewise, our findings revealed an increase in TGF-β and MMP-9 in the D-GAL-injected groups, specifically in males, with an associated prominent structural vascular remodeling and interstitial fibrosis in cardiac microscopy. However, eugenol prevented cardiac hypertrophy and fibrosis in many previous studies by downregulating TGF-β as in cardiomyopathy in streptozotocin-induced diabetic rats and chlorpyrifos-induced cardiotoxicity (Parsa-Lisar et al. 2022; Qar et al. 2022). Concomitantly, TGF-β and MMP-9 were significantly downregulated in eugenol- and nano-eugenol-treated groups in this study, with a subsequent decrease in cardiac fibrosis% as indicated by picrosirius staining compared to D-GAL-treated rats. Thus, eugenol and its nano-emulsion preserved the cardiac structure nearly normal, prevented cardiac dilatation and vascular remodeling, and maintained cardiac function when administered concurrently with D-GAL.

This study revealed severe cardiac structural alterations, collagen deposition, and fibrosis in aged male rats compared to females. Moreover, oxidative stress, inflammation, and mitophagy-related factors were higher in aged male rats than in female ones. A significant drop in cardiac SIRT1 level was depicted in male rats compared to females in the D-galactose-induced group. Consequently, males were more susceptible to developing cardiac complications with aging than females, which align with some studies that outlined that the animals’ systolic function decreased with age in males but not in females, and male myocytes are more susceptible to apoptosis than female myocytes, although both genders have the same number of myocytes at birth (Koch et al. 2013; Senyo et al. 2014). Although the estrogen level was not evaluated in this study, it was reported previously to exert a well-recognized protective effect on the cardiovascular system by reducing inflammation and oxidative stress (Xiang et al. 2021). Further, eugenol nano-emulsion exerted significant ameliorative effects against D-galactose-induced aging than its native form, which may be attributed to increased bioavailability of eugenol through nanotechnology.

## Conclusion

This study demonstrates that oral eugenol and its nano-emulsion confer cardioprotective effects in a D-galactose-induced aging model, mitigating oxidative stress, mitochondrial dysfunction, and cardiac tissue damage. Notably, male rats showed greater vulnerability to cardiac impairment than females, underscoring the importance of sex-specific investigations. While the nano-emulsion exhibited promising benefits, further work is needed to establish its comparative advantage over free eugenol and to clarify the molecular basis of gender-related differences. Overall, these findings position eugenol nano-emulsion as a potential candidate for addressing age-associated cardiac complications, warranting deeper exploration in future studies.

## Supplementary Information

Below is the link to the electronic supplementary material.

ESM 1 DOCX (486 KB)

## Data Availability

All the data will be available upon reasonable request.
