# Supplementary material for: The role of mitochondrial dysfunction, oxidative stress, and gender in cardiac fibrosis and vascular remodeling in an induced aged rat model with possible mitigation by eugenol nano-emulsion
Source: Naunyn Schmiedebergs Arch Pharmacol. 2026 Apr 13;399(9):14203–19. doi: 10.1007/s00210-026-05262-4 (PMC13357453; doi:10.1007/s00210-026-05262-4)
Supplement: Supplementary file 1 — DOCX (486 KB) [file 210_2026_5262_MOESM1_ESM.docx]

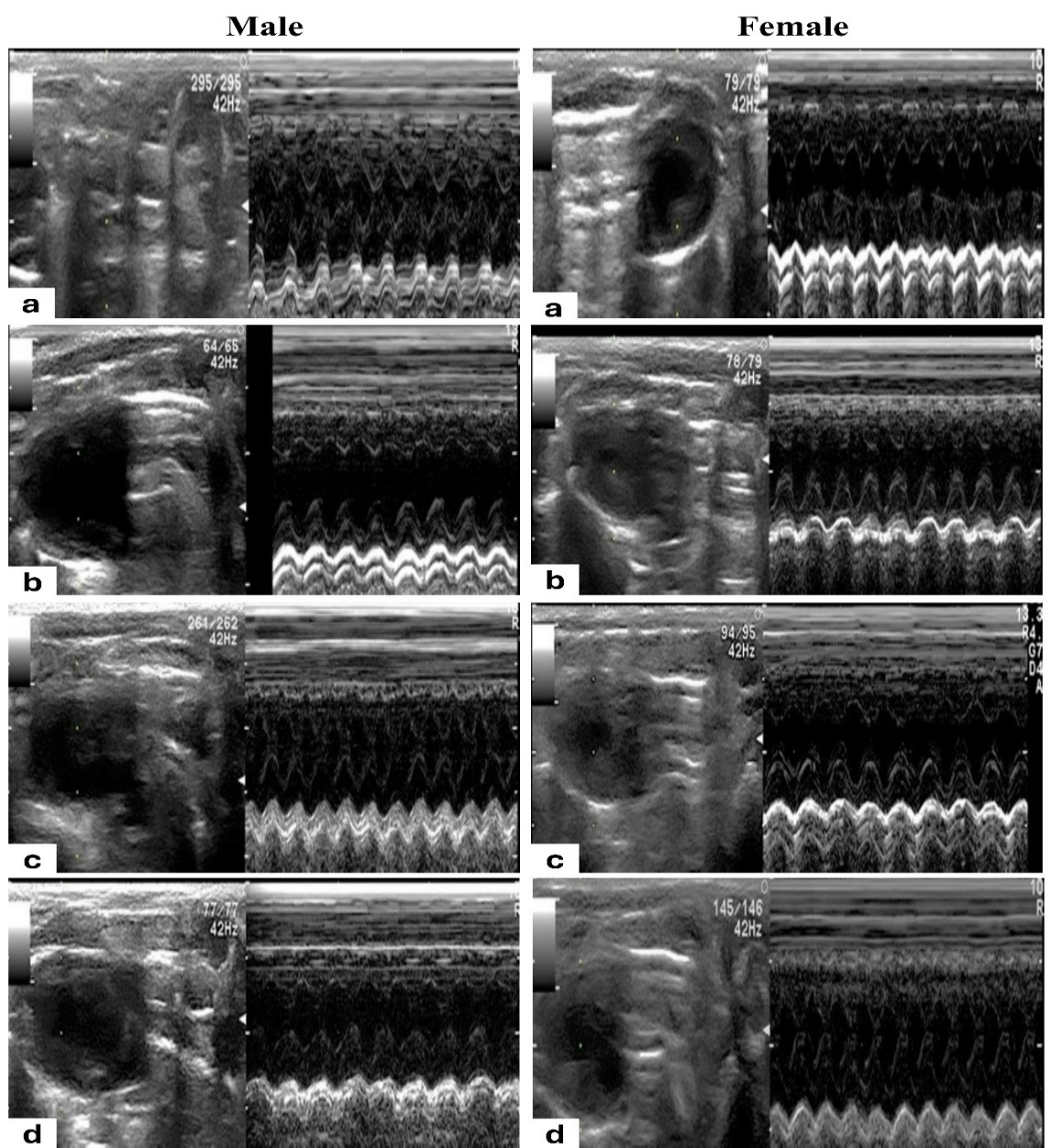
**Fig. 1S**. Representative images of M-mode tracing of echocardiography, (a) negative control male and female groups. (b) D-GAL male and female injected groups. (c) D-GAL+eugenol male and female treated groups. (d) D-GAL+nano-eugenol male and female treated groups
